# Healthcare professionals' intentions to use clinical guidelines: a survey using the theory of planned behaviour

**DOI:** 10.1186/1748-5908-5-51

**Published:** 2010-06-29

**Authors:** Tiina Kortteisto, Minna Kaila, Jorma Komulainen, Taina Mäntyranta, Pekka Rissanen

**Affiliations:** 1 Tampere School of Public Health, University of Tampere, Medisiinarinkatu 3, Tampere, Finland; 2 City of Tampere, Social and Primary Care Services/Children and Youth Health Services, Tampere, Finland; 3 National Institute for Health and Welfare, Mannerheimintie 166, Helsinki, Finland; 4 The Finnish Medical Society Duodecim, Kalevankatu 11A, Helsinki, Finland; 5 The Ministry of Social Affairs and Health, Meritullinkatu 8, Helsinki, Finland

## Abstract

**Background:**

Finnish clinical guidelines are evolving toward integration of knowledge modules into the electronic health record in the Evidence-Based Medicine electronic Decision Support project. It therefore became important to study which factors affect professionals' intention to use clinical guidelines generally in their decision-making on patient care. A theory-based approach is a possible solution to explore determinants of professionals' behaviour. The study's aim was to produce baseline information for developers and implementers by using the theory of planned behaviour.

**Methods:**

A cross-sectional internet-based survey was carried out in Finnish healthcare organisations within three hospital districts. The target population (n = 2,252) included physicians, nurses, and other professionals, of whom 806 participated. Indicators of the intention to use clinical guidelines were observed by using a theory-based questionnaire. The main data analysis was done by means of multiple linear regressions.

**Results:**

The results indicated that all theory-based variables--the attitude toward the behaviour, the subjective norm, and the perceived behaviour control--were important factors associated with the professionals' intention to use clinical practice guidelines for their area of specialisation in the decisions they would make on the care of patients in the next three months. In addition, both the nurse and the physician factors had positive (p < 0.01) effects on this intention in comparison to other professionals. In the similar models for all professions, the strongest factor for the physicians was the perceived behaviour control, while the key factor for the nurses and the other professionals was the subjective norm. This means that context- and guideline-based factors either facilitate or hinder the intention to use clinical guidelines among physicians and, correspondingly, normative beliefs related to social pressures do so for nurses and other healthcare professionals.

**Conclusions:**

The results confirm suggestions that the theory of planned behaviour is a suitable theoretical basis for implementing clinical guidelines in healthcare practices. Our new finding was that, in general, profession had an effect on intention to use clinical guidelines in patient care. Therefore, the study reaffirms the general contention that different strategies need to be in place when clinical guidelines are targeted at different professional groups.

## Background

Clinical guidelines are systematically developed to assist healthcare professionals and patients in making treatment decisions [1]. In Finland, there are long traditions of developing national electronic guideline databases [2]. These are used via a national health portal http://www.terveysportti.fi throughout the healthcare system (in all primary care centres and secondary care hospitals) [3]. Clinical guidelines seem well disseminated to healthcare organisations, but there is still scant evidence on adoption in clinical practice [4-7].

There are several obstacles to guideline adherence, some of which are related to the professionals, such as lack of awareness, agreement, self-efficacy, and inertia of previous practice. There are also guideline-, patient-, and environmental-related barriers that are influenced further by context [8]. For successful implementation of guidelines, there is a need to better understand the complexity of changing clinical practice and especially the implementation problems that relate to professional attitudes and experiences associated with use of guidelines in the healthcare context [9-12].

**Figure 1:**
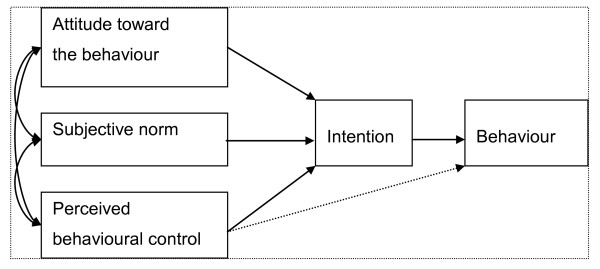
**A framework of the theory of planned behaviour **[17]**; see also **[63].

A theory-based approach is a possible solution for exploring determinants of professionals' behaviour [13,14]. The theory of planned behaviour (TPB) is a conceptual framework for understanding human social behaviour [15,16]. The TPB states that one central determinant of behaviour is an intention to perform it. The strength of intention consists in three kinds of latent components (see Figure 1). The first component, the attitude toward the behaviour, is composed of human beliefs about consequences of the behaviour. The second component, the subjective norm, is composed of human normative beliefs and social pressure toward the behaviour. The third component, the perceived behaviour control, is composed of human beliefs concerning capability and the controllability of performing the behaviour. The latter can also be directly associated with the behaviour [17].

Applying the TPB to identify which theoretical constructs predict use of guidelines in clinical practice, as has been done in studies among healthcare professionals [18,19], is advisable since intention seems to be a valid proxy measure for behaviour [20]. These studies have targeted either a specific profession, such as gynaecologists [21], or one specific guideline in a specific healthcare setting--for example, hand hygiene among neonatal healthcare workers [22]. One American study [23] examined physicians' compliance with one of four specific sets of clinical guidelines on five practice sites--for example, an asthma guideline among two family practice residency groups. The results show that the perceived behavioural control was the strongest predictor of physicians' behavioural intention.

However, there are still some concerns about the correspondence between an intention and a future behaviour, particularly in healthcare professionals' practice [24], even though intentions explaining 28% of the variance of behaviour should be considered 'good' [25]. In addition, both nurses' and physicians' self-reported adherence to guidelines have been assessed as greater than their actual use of guidelines [23,26]. Moreover, in a systematic review [19], it was shown that a number of methodological and theoretical moderators may influence the efficacy of prediction of intention.

This study focuses on the general level of clinical guidelines' use in healthcare practice wherein each profession has its own duty concerning, and also relationship to, guidelines. Only a few previous studies applying qualitative methods [27-29] have explored this topic before, none of them in a Finnish context. Here, the TPB-based survey is applied for the first time among several types of professionals (physicians, registered nurses, public health nurses, midwives, ward nurses, physiotherapists, occupational therapists, and others) in both primary and secondary care. In addition, Finnish clinical guidelines are currently evolving from access via the internet environment to integration of knowledge modules into the electronic health record in the Evidence-Based Medicine electronic Decision Support (EBMeDS) project [30-32]. To produce baseline information, it was considered important to study which factors affect professionals' intention to use clinical practice guidelines in making their decisions concerning care of patients. The study questions were as follows: Do healthcare professionals have positive or negative intention to use clinical guidelines for their area of specialisation in their decision-making for patient care? How do healthcare professions differ in their intentions? What factors are associated with healthcare professionals' intention to use clinical guidelines in patient care?

## Methods

### Procedures and participants

A cross-sectional internet-based survey was carried out from October 2006 to May 2007 in publicly funded healthcare organisations (n = 26) within three hospital districts, which were to become the pilot sites of EBMeDS. The target population included all physicians, registered nurses, and other healthcare professionals with at least nursing-level education in the Kymenlaakso (KL) and Central-Finland (CF) hospital districts (n = 1,400); units of dental care, radiology, and laboratory workers were excluded. In the hospital district of Northern-Savo (NS), professionals involved in the care of diabetes were included (n = 913). Different professions were included because the EBMeDS system was to be piloted among all of these groups. The target groups were approached through a contact person nominated from the participating organisations, the chief medical officers of which approved the study.

The final target study population consisted of 2,252 professionals (61 professionals were excluded because of, for example, an invalid e-mail address). After two reminders, 806 healthcare professionals responded: 135 physicians (out of 463), 552 nurses (out of 1,477), and 112 other professionals (out of 312).

### Questionnaire

A questionnaire was designed by the EBMeDS study group complemented by two advisers with psychology degrees. The aim was to develop a multifaceted and practical questionnaire consisting of relevant questions. Therefore, the first two questionnaires were constructed to be of differing length. These were piloted among a convenience sample of healthcare professionals (n = 56) randomised into two groups [33]. Pilot group one were given the longer questionnaire one, and pilot group two received the shorter version two, in an internet-based survey. The response rate increased from 22% to 44% in group one, and from 36% to 50% in group two after one reminder. The respondents gave valuable feedback, such as that questionnaire one was too long, questions were targeted more to physicians than nurses, there were too many issues addressed within one question and by the questionnaire overall, and formulation of a very informative covering letter would encourage responses.

Next, JK and TK carefully considered each question in relation to the objectives of the EBMeDS project. The EBMeDS study group reflected on the feedback and then abbreviated the questionnaire to 27 questions in the following areas: information technology questions, which included nine questions about the usefulness of and satisfaction with the electronic patient record and information databases; guideline questions, which involved the Attitudes towards Guidelines Scale [34] and included also the TPB-based items; job content questions, which applied a concise form of the Job Content Questionnaire [35]; and questions on the respondent's individual and organisational background. Four investigators tested the technical validity of the internet questionnaire. Here, we included the TPB-based items and background questions (see Additional File 1). A covering letter described the objectives of the study, with a link to the web pages of the EBMeDS project, approval of the study, and investigator information [33].

### Indicators

The items in the guideline-based set of questions were designed according to the principles of the brief form of the TPB questionnaire manual [36]. In keeping with the principle of compatibility [17,37,38], the four indicators referred to clinical practice guideline use in general, not one specific guideline. The target behaviour is considered to involve a professional's knowing use of patient-specific guidelines in clinical decision-making, which was not directly observed. The dependent variable was an intention, which was measured with one item: 'I intend to use clinical practice guidelines for my area of specialisation in the decisions I make on the care of patients in the next three months.' The first latent component, the attitude toward the behaviour, was assessed by way of three behaviour beliefs associated with the use of clinical practice guidelines. The second latent component, the subjective norm, was assessed in terms of three normative beliefs about social pressures to use clinical practice guidelines. The third latent component, the perceived behaviour control, was assessed with six control beliefs about context and guideline factors that might facilitate or hinder use of clinical practice guidelines. These behavioural, normative, and control belief items were developed by means of a manual [36], earlier evidence [39,40], and guideline-based Finnish national document [41] such that each of them should be relevant and important for healthcare professionals in the Finnish healthcare context. Each item for the variables was assessed directly by the respondent, rated on a seven-point scale: 1 = absolutely negative, 2 = negative, 3 = probably negative, 4 = neither negative nor positive, 5 = probably positive, 6 = positive, 7 = absolutely positive.

### Analyses

Statistical analyses were performed with SPSS for Windows, version 15.0. The characteristics of the sample and the dependent variable frequency were analysed with descriptive statistics. Factor analysis with principal component analysis, using the varimax rotation method, was carried out for 12 TPB items in order to verify the discriminant validity of the three predicted variables computed in the analysis [42]. These items were combined according to the theory into three latent components. The internal consistency of the scales, measured via Cronbach's alpha coefficient, was above 0.8 for each of these variables, which can be considered a satisfactory value [43]. Profession group differences for the intention variable were analysed via variance analysis with Welch's and Gamess-Howell's tests, which have been recommended for use in cases of unequal sample sizes and unequal variances [44]. The main data analyses were conducted with multiple linear (ordinary least square) regressions [45]. The models were formed to use the theory-based variables, dummy variables related to respondents (age and gender) and profession in the overall model, and organisation characteristics (healthcare level and hospital district). In the analyses, the variables were directly entered in the model to investigate the effect of each on the professionals' intention to use clinical practice guidelines. The theory-based TPB variables were handled as continuous in the models despite being composed of only seven discrete values. Subjects with missing values were excluded from all analyses. This caused a reduction in the number of respondents, which is reported upon further in the discussion section.

## Results

The e-mail invitation to participate the internet-based survey was followed by two reminders. The overall response rate was 36%; broken down by profession, it was 29% among physicians, 37% for nurses, and 36% for other professions. The majority of the respondents (89%) were female (see Table 1), and the mean age was 45 years (range: 24 to 67 years). The distribution by profession was 17% physicians, 69% nurses (registered nurses, public health nurses, and midwives), and 14% other professionals in the healthcare field (physiotherapists, ward nurses, occupational therapists, rehabilitation advisers, *et al.*).

**Table 1:** Characteristics of the respondents, compared to the target population

	Respondents		Target	
	**n**	_**%**_	**n**	_**%**_

**Gender (n = 792)**				
Female	703	89	1,948	87
Male	89	11	304	13
**Age (n = 788)**			No information	
Below 35 years	103	13		
35 to 44 years	258	33		
45 to 54 years	327	41		
55 and over	100	13		
**Profession (n = 799)**				
Physician	135	17	463	20
Nurse	552	69	1,477	66
Other	112	14	312	14
**Healthcare level (n = 799)**				
Primary care	437	55	1,105	49
Secondary care	362	45	1,147	51
**Hospital district (n = 802)**				
KL^a^	423	53	1,248	55
NS^b^	326	40	888	40
CF^c^	53	7	116	5

^a^KL = Kymenlaakso hospital district.

^b^NS = Northern-Savo hospital district.

^c^CF = Central-Finland hospital district.

The intention to use clinical practice guidelines in decision making for patient care was more often positive than negative. Overall, 18% of the respondents indicated absolutely positive and 30% positive intention, while only 1% indicated absolutely negative and 4% negative views. The mean score of the intention variable was 5.5 points for the physicians, 5.3 for the nurses, and 5.0 for the other professionals (see Table 2). The Welch's variance-weighted ANOVA (asymptotically F 3.83, p = 0.02) indicated that at least one difference existed between the groups. Further, the Games-Howell's test indicated positive differences between physicians and nurses (mean difference 0.30, p = 0.04), and between physicians and other professionals (mean difference 0.42, p = 0.04).

**Table 2:** Description of the variables in the models--means (standard deviations)

Variable	Overall model	Physicians model	Nurses model	Others model
	(n = 638)	(n = 123)	(n = 436)	(n = 79)
Theory of planned behaviour variables				
Intention (1 item), scale: 1-7^a^	5.3 (1.3)	5.5 (1.2)	5.3 (1.3)	5.0 (1.3)
Attitude (3 items), scale: 1-7^a^	5.4 (1.2)	5.2 (1.3)	5.4 (1.2)	5.4 (1.2)
Subjective norm (3 items), scale: 1-7^a^	5.4 (1.0)	5.4 (1.0)	5.4 (1.1)	5.4 (1.0)
Perceived behaviour control (6 items), scale: 1-7^a^	4.4 (0.8)	4.5 (0.8)	4.3 (0.9)	4.4 (0.8)

Individual-level variables for the respondents				
Gender (male = 0, female = 1)	0.9 (0.3)	0.6 (0.5)	0.9 (0.2)	0.9 (0.2)
Age	44.4 (8.4)	45.8 (8.9)	43.9 (8.3)	45.2 (7.7)
Nurse_d (nurse = 1, physician or other professional = 0)	0.7 (0.5)	-	-	-
Physician_d (physician = 1, nurse or other professional = 0)	0.2 (0.4)	-	-	-

Organisation-level variables for the respondents				
Primary care (primary care = 1, secondary care = 0)	0.6 (0.5)	0.5 (0.5)	0.6 (0.5)	0.7 (0.5)
KL (KL = 1, others = 0)^b^	0.5 (0.5)	0.4 (0.5)	0.5 (0.5)	0.5 (0.5)
NS (NS = 1, others = 0)^b^	0.4 (0.5)	0.5 (0.5)	0.4 (0.5)	0.4 (0.5)

^a ^Higher score = more positive view.

^b ^Kymenlaakso (KL) and Northern-Savo (NS) hospital districts, as dummy variables, with the Central-Finland hospital district (CF) as a reference group in the regression models.

The factors associated with the professionals' intention to use clinical practice guidelines were analysed via multiple linear regression models. The overall regression model was statistically acceptable (F = 37.41, p < 0.001) and explained 36% (adjusted R square) of the variation in the intention to use clinical guidelines. Moreover, the TPB variables, as well as nurse and physician variables, had a positive effect on the intention to use clinical practice guidelines (see Table 3). When similar models were run in both primary and secondary care settings, the positive profession effect on the intention remained among secondary care workers (B = 0.55, p = 0.01 among nurses and B = 0.98, p < 0.001 among physicians) but did not remain statistically significant among primary care workers. After these results, similar regression models were run in each profession group.

**Table 3:** Effects of TPB variables and individual and organisation characteristics on professionals' intention to use clinical guidelines--ordinary least squares models

	Overall model			Physicians model			Nurses model			Others model		
Variables	B	t-test		B	t-test		B	t-test		B	t-test	
Constant	0.84	2.07	*	1.15	1.26		0.98	2.03	*	1.96	1.50	
Attitude	0.26	6.15	***	0.24	3.11	**	0.27	5.18	***	0.15	1.16	
Subjective norm	0.34	7.13	***	0.27	2.67	**	0.33	5.71	***	0.48	3.02	**
Perceived behaviour control	0.34	5.94	***	0.45	3.82	***	0.28	3.99	***	0.35	2.03	*
Gender	-0.01	-0.09		0.01	0.05		0.09	0.38		-0.07	-0.12	
Age	-0.01	-1.62		-0.01	-1.19		-0.00	-0.54		-0.03	-1.84	
Primary care	0.9	0.93		-0.40	-2.34	*	0.20	1.74		0.30	0.97	
KL^a^	-0.32	-1.89		0.07	0.16		-0.24	-1.14		-0.71	-1.63	
NS^a^	-0.18	-1.11		0.45	1.10		-0.21	-1.03		-0.55	-1.33	
Nurse_dummy^b^	0.34	2.70	**									
Physician_dummy^b^	0.52	3.25	**									

n	637			122			435			78		
R square	0.37			0.52			0.36			0.39		
Adjusted R	0.36			0.48			0.34			0.32		
F	37.41		***	15.13		***	29.73		***	5.56		***

***p < 0.001, **p < 0.01, *p < 0.05

^a ^Kymenlaakso (KL) and Northern-Savo (NS) hospital districts: dummy variables, with the Central-Finland hospital district (CF) as a reference group.

^b^The other profession group was a reference group in the overall model.

The physicians model explained 48% variation in the intention to use clinical guidelines (see Table 3). All TPB variables were positively correlated with the intention variable. The strongest of these was perceived behaviour control, showing a positive association with the intention variable. This indicates that the physicians, who had a more positive view of contexts and guideline factors, also intended to use clinical practice guidelines more often. Among the variables of individual and organisation characteristics, only the variable for primary care had a negative effect on the intention variable, thus showing less intention among primary care physicians to use clinical practice guidelines than among secondary care physicians.

The nurses model explained 34% of the variation in the intention to use clinical guidelines (see Table 3). Of all variables in the model, only the TPB variables were positively correlated with the intention variable. The subjective norm was the strongest factor, indicating that those nurses who perceived social pressure to use clinical practice guidelines also had more positive intention to use them than did nurses who did not perceive social pressure.

The model for other professionals explained 32% of the variation in the intention to use clinical guidelines (see Table 3). Of all variables, only the subjective norm and the perceived behaviour control were positively correlated with the intention variable. The subjective norm was the strongest factor, indicating that the professionals' perception of social pressure toward the use of clinical guidelines produced positive intention to use them.

## Discussion

### Main results

The results of this study indicate that the TPB variables--the attitude toward the behaviour, the subjective norm, and the perceived behaviour control--are important factors associated with the healthcare professionals' intention to use clinical practice guidelines generally in their decisions on patient care. Consequently, the results confirm suggestions that the TPB is a suitable theoretical basis for implementation of clinical guidelines in multiple healthcare professions' practices [13,20,46].

An important finding for clinical guideline developers and implementers is that both the nurses and the physicians had stronger intention to use clinical guidelines in patient care than other professionals did when other factors in the model were fixed. In particular, this effect was strong among secondary care workers. On the other hand, nurses and physicians had similar intention to utilise clinical guidelines when compared only against each other in a regression model. Thus, our results indicate that contextual factors, such as multiple profession groups or healthcare setting, were important in our model.

In the profession-based models, the factor associated most strongly with intention was the perceived behaviour control for the physicians, but the subjective norm for the nurses and other professionals. These results indicate that, in particular, context- and guideline-based factors either encourage or hinder the intention to use clinical practice guidelines among physicians, and that normative beliefs related to social pressures have a corresponding effect for nurses and other professionals. It can be argued that for successful implementation of clinical guidelines the implementers should recognise and make better use of those context and guideline factors that can have a positive effect on implementation by physicians as well as those normative belief factors with positive effects, such as a superior's support for use of clinical guidelines, for nurses and other professionals [47]. According to the behaviour science perspective [15,16,38], it is necessary at the first stage in planning of an implementation to identify the beliefs behind the target behaviour where one wishes to see change.

Similar findings to those for the physician group have been reported earlier [23,39,48,49]. However, also opposite results have been reported; for example, Puffer and Rashidian [40] found that among nurses the attitude toward the behaviour and the perceived behaviour control are the most important indicators of intention to offer smoking cessation advice. Limbert and Lamb [50] found the subjective norm the strongest indicator of intention to use the asthma guidelines and the attitude toward the behaviour the strongest indicator of intention to use the antibiotic guidelines among physicians. However, these differences from our results could be simply explained by the different target behaviour. This study considered not specific guideline-based behaviours but, instead, professional's general self-reported behaviour in the patient-specific use of guidelines.

The variables of individual and organisation characteristics had no effect or only a modest one on the professionals' intention to use clinical practice guidelines in the profession-based models. The negative effect of the primary care variable in the physicians group may be clinically relevant, highlighting the nature of the work environment for guideline implementers. This phenomenon is described thoroughly by McKenna *et al. *[51], who analysed studies of barriers to evidence-based practice in primary care. The conclusions were that potential barriers to target behaviour have to be identified specifically in relation to the work environment in which they arise, and that there was only limited high-quality evidence available of this phenomenon.

We found that the intention to use clinical practice guidelines in decision making regarding patient care was, for the most part, positive for all professions. Almost one-half of the respondents had positive intentions, and only 5% were negative. This is a positive message for implementation of the EBMeDS in clinical practice. It also confirms our earlier findings among Finnish physicians [52]. It seems that there exist in Finland potential pilot users for automatic reminders based on the clinical guidelines. However, it is equally important to notice that 40% of physicians and 50% of nurses and other professionals responded with a 3, 4, or 5 on the seven-point scale here. It seems that the main conclusion is that almost one-half of the respondents were uncertain of their intentions or that intentions may change in changing clinical situations. Another possibility is that the intention item 'I intend to use clinical practice guidelines for my area of specialisation in the decisions I make on the care of patients in the next three months' was too general, and therefore it was hard for professionals to respond more precisely. This, in turn, may simply translate into tailoring behaviour individually according to the patients' needs. These findings are in line with previous evidence on the use of guidelines in Finnish primary care [53,54] and secondary care [7]. For example, the guidelines concerning resuscitation are reported to be used in only 42% of Finnish health centres [5].

Differences were found in the variance analysis between professions in their intentions to use clinical guidelines. The score for this intention was higher among physicians than among nurses or other professionals. Similar results were reported in the study of Goossens's *et al. *[55], wherein physicians' and nurses' willingness to adopt a set of guidelines at an academic medical centre were compared. This is an important message for overcoming possible barriers in implementation of the EBMeDS in a multi-profession context. An Australian study [56] also found that education of professionals and motivation of multidisciplinary groups to redesign care processes can aid in overcoming potential barriers to implementation. In addition, our results reaffirm that needs of nurses and other professionals have to be carefully targeted in the development of automatic reminders for those specific groups [57].

### Strengths and limitations

The strength of the study lies in its comprehensiveness: in contrast to previous studies [19] here all major healthcare professions, in both primary and secondary care, were represented. Also, the study concentrated on factors that possibly can affect professionals' intention to use clinical guidelines in their decision making. The choice of factors was based on the TPB and on previous findings [13,15,17,20,23,40]. A recent systematic meta-review of factors influencing implementation of clinical guidelines for healthcare professionals listed factors such as characteristics of the guidelines, professionals, patients, and environment that influence use of guidelines [58]. Another review highlighted that evidence concerning proxy measures of clinicians' behaviour is still limited [59].

The study design was a cross-sectional survey at the EBMeDS pilot sites. These results are utilised in system development and testing. In comparison of the respondents to the target population (Table 1), it seems that the participants are representative in their gender, profession, and hospital district. In spite of this, only a small difference (6%) was found between healthcare levels. This could be a potential source of bias in the interpretation of the study results.

A clear limitation is the low response rate and the missing values for some of the respondents. These may cause non-response bias and, accordingly, problems in interpretation of the results [45]. Since an internet-based webropol format was used, the response rate can be assessed by using the work of Bosnjak *et al. *[60]: of a total of 2,252 potential respondents, 47% did not open the questionnaire, 14% viewed the questionnaire (*i.e.*, opened the web link in their e-mail letter) but did not start to respond, 5% began to respond but did not complete the questionnaire, and 36% responded. At least two reasons can be posited to explain the low response rate. First, the busy healthcare workers may have felt that they did not have enough time to complete the survey and the covering letter did not convince them of the need to do so. Second, the internet-based survey and questions may have been too technically difficult or unusual for some. Recent evidence on surveys of healthcare professionals supports these assumptions [61,62].

By profession, 9% of the physicians' responses had missing values and were therefore excluded from the analyses; the corresponding figure was 21% for nurses and 29% for other professionals. Accordingly, the real response rates in the regression models were 26% for physicians, 29% for nurses, and 25% for other professionals. Although the variance explaining the intention (R square), at above 28%, can be classed as good [25], the interpretations of the other professionals' regression model (adjusted R square 0.32, F value 5.56) cannot be practically generalised, because the results came from a low total number of respondents (n = 79), who, in addition, represented many, different professions. However, the applicability of the results in the physicians' and nurses' groups is rather good--the variables of the physicians' model explained 48% (adjusted R square 0.48, F value 15.13) of the variation in the intention to use clinical guidelines, and the 436 nurses were representative clinical guideline users of all relevant nursing professions.

Our main target in the formulation of the questionnaire was the unique EBMeDS study context concerned, not more general approaches [33,45]. However, a theory-based approach was used in this formulation [36], and the questionnaire was piloted and refined on the basis of the findings from the pilot tests [33]. These actions confirmed the content validity of the questionnaire. Similarly, the internal consistency of the sum variables was analysed as being adequate (Cronbach's alpha coefficient over 0.8 for each variable). We only used one item related to the intention variable, which can be considered a methodological limitation [17,36] (see Additional File 1). In subsequent analyses, from the same study context, in different survey data (n = 38 primary care professionals' responses), we tested the extent of the correlation between a single intention variable ('I intend to do...') and a generalised intention variable ('I except/want/intend to do...') [36]. We found that the single intention variable explained 82% (adjusted R square 0.82, F = 164.36, p < 0.001) of the variation of the generalised intention variable. Thus, we acknowledge a methodological limitation of our questionnaire formulation, but this potential source of bias seems minor. Finally, it has to be recognised that the results of this study are based on the professionals' self-reported assessments, which were not verified with observations of actual use of clinical guidelines [23,26].

## Summary

Regardless of some limitations of our study, we conclude that we found some support for the idea of using TPB for implementation of clinical practice guidelines in multiple professional groups. The new finding that is of importance for guideline developers and implementers is that, when compared to other professionals, both nurses and physicians had positive intention to use clinical practice guidelines in patient care. This reaffirms the general contention that different strategies need to be in place in targeting of different professional groups. It could be worth investigating whether involving the various groups more intensively from the beginning of guideline development all the way through to implementation, or supporting guideline uptake, would have a positive effect on adoption in their decision making.

## Competing interests

The authors declare that they have no competing interests.

## Authors' contributions

All authors conceived the study and designed the questionnaire. TK, MK, and PR were responsible for data collection. TK analysed the data, and PR supervised the analyses. TK led the writing process, and all authors commented on sequential drafts and approved the final version of the manuscript.

## Supplementary Material

Additional file 1 **The guidelines and background questions PDF**. Click here for file
