# Supplementary material for: Healthcare professionals' intentions to use clinical guidelines: a survey using the theory of planned behaviour
Source: Implement Sci. 2010 Jun 29;5:51. doi: 10.1186/1748-5908-5-51 (PMC2902417; doi:10.1186/1748-5908-5-51)
Supplement: Additional file 1 — The guidelines and background questions PDF. [file 1748-5908-5-51-S1.PDF]

Additional file 1: Questions on clinical practice guidelines and respondent’s background

# Questions on clinical practice guidelines and respondent’s background

**1. I intend to use clinical practice guidelines for my area of specialisation to the decisions I make on the care of patients in the next three months.**

| | | | | | | | | |
|---|---|---|---|---|---|---|---|---|
| Definitely not | 1 | 2 | 3 | 4 | 5 | 6 | 7 | Definitely yes |

**2. To what extent do you agree or disagree with the following propositions?**
**By following clinical practice guidelines in my clinical decision-making …**

| | I totally disagree | | | | | | I fully agree |
|---|---|---|---|---|---|---|---|
| a. I offer my patients higher-quality care | 1 | 2 | 3 | 4 | 5 | 6 | 7 |
| b. I ensure that all my patients obtain the same level of basic care | 1 | 2 | 3 | 4 | 5 | 6 | 7 |
| c. I am able to contribute to controlling the costs of care | 1 | 2 | 3 | 4 | 5 | 6 | 7 |

**3. How do the following people regard the fact that you use clinical practice guidelines for your area of specialisation in your clinical decision-making?**

| | Definitely should not use | | | | | | Definitely should use |
|---|---|---|---|---|---|---|---|
| a. Patients | 1 | 2 | 3 | 4 | 5 | 6 | 7 |
| b. My supervisor | 1 | 2 | 3 | 4 | 5 | 6 | 7 |
| c. My colleagues (doctors/nurses) | 1 | 2 | 3 | 4 | 5 | 6 | 7 |

**4. To what extent do the following factors prevent or facilitate your use of guidelines applicable to your area of specialisation?**

| | | Prevents to a great extent | Markedly prevents | Slightly prevents | Has no effect | Slightly facilitates | Markedly facilitates | Facilitates to a great extent |
|---|---|---|---|---|---|---|---|---|
| a. | Lack of time | 1 | 2 | 3 | 4 | 5 | 6 | 7 |
| b. | Clarity and understandability of the guidelines | 1 | 2 | 3 | 4 | 5 | 6 | 7 |
| c. | Inflexibility of the guidelines (i.e., they may not take into account the needs of individual patients) | 1 | 2 | 3 | 4 | 5 | 6 | 7 |
| d. | The guidelines incorporating the viewpoints of my professional group | 1 | 2 | 3 | 4 | 5 | 6 | 7 |
| e. | The guidelines being easily accessible | 1 | 2 | 3 | 4 | 5 | 6 | 7 |
| f. | The guidelines being based on scientific evidence | 1 | 2 | 3 | 4 | 5 | 6 | 7 |

## Background questions

**1. Gender**

1 male
2 female

**2. Age** ______ years

**3. Profession**

1 physician
2 nurse
3 community nurse
4 physiotherapist
5 other – which: ____________________________________

**4. What health care level do you work in?**

1 primary health care (general practice)
2 other primary health care
3 secondary or tertiary health care
4 other – which: _____________________________________________

**5. What hospital district do you work in?**

1 Kymenlaakso
2 Northern Savo
3 Central Finland
